# High fat and/or high salt intake during pregnancy alters maternal meta‐inflammation and offspring growth and metabolic profiles

**DOI:** 10.14814/phy2.12110

**Published:** 2014-08-05

**Authors:** Clare M. Reynolds, Mark H. Vickers, Claudia J. Harrison, Stephanie A. Segovia, Clint Gray

**Affiliations:** 1 Liggins Institute and Gravida, National Centre for Growth and Development, University of Auckland, Auckland, New Zealand

**Keywords:** Developmental programming, high fat, high salt, inflammation, maternal metabolism

## Abstract

A high intake of fat or salt during pregnancy perturbs placental function, alters fetal development, and predisposes offspring to metabolic disease in adult life. Despite its relevance to modern dietary habits, the developmental programming effects of excessive maternal fat and salt, fed in combination, have not been examined. We investigated the effects of moderately high maternal fat and/or salt intake on maternal metainflammation and its consequences on fetal and weanling growth and metabolic profile. Female Sprague–Dawley rats were fed a standard control diet (CD), 4% salt diet (SD), 45% fat diet (HF) or 4% salt/45% fat combined diet (HFSD) 3 weeks prior to and throughout pregnancy and lactation. Plasma and tissue samples were collected at day 18 of pregnancy from mother and fetus, and at postnatal day 24 in weanlings. Markers of adipose tissue inflammation, macrophage infiltration, lipogenesis, nutrient transport, and storage were altered in pregnant dams receiving high‐fat and/or ‐salt diets. This was accompanied by increased fat mass in high‐fat groups and differential hepatic lipid and glucose homeostasis. Offspring of high fat‐fed mothers had reduced fetal weight, displayed catch‐up growth, increased fat mass, and altered metabolic profiles at weaning. Maternal diets high in fat and/or salt affect maternal metabolic parameters, fetal growth and development, metabolic status, and adipoinsular axis in the weanling. Results presented here highlight the importance of diet in expectant mothers or women considering pregnancy. Furthermore, the potential for maternal nutritional intervention strategies may be employed to modify the metabolic disease risk in adult offspring during later life.

## Abstract (precis)

We investigated the effects of moderately high maternal fat and/or salt intake on maternal metainflammation and its consequences on fetal and weanling growth and metabolic profile. Maternal diets high in fat and/or salt affect maternal metabolic parameters, fetal growth and development, metabolic status, and adipoinsular axis in the weanling. Results presented here highlight the importance of diet in expectant mothers or women considering pregnancy.

## Introduction

The links between obesity, insulin resistance, and immune function have provided the basis for the emerging field of immunometabolism which implicates immune cell infiltration and increased expression of inflammatory mediators such as TNF*α* and IL‐1*β* in the pathophysiology of metabolic disease (Ahima 2011). The low‐grade inflammatory phenotype observed in obesity and associated comorbidities may be crucial for understanding the increased incidence in pregnancy‐related disorders such as gestational diabetes and preeclampsia (Bodnar et al. 2005; Chu et al. 2007) in overweight/obese women of childbearing age (Challis et al. 2009; Hillemeier et al. 2011; Nodine and Hastings‐Tolsma 2012). This study investigates the contribution of maternal high‐fat and/or ‐salt diet on the maternal metainflammatory phenotype both systemically and locally within the adipose tissue.

There has been an exponential increase in overweight/obesity rates worldwide, with as many as one in three adults now displaying overweight or obese phenotypes (Berghofer et al. 2008). Although increased rates of obesity and related metabolic disorders have largely been attributed to lifestyle factors such as Western‐style diets and sedentary activity, there is also increasing evidence that an altered early life environment is a major risk factor for the development of obesity and metabolic dysfunction in later life. The Western diet is moderately high in both saturated fat and salt, with a high fat intake usually paralleled by a high salt intake (O'Keefe and Cordain 2004; Cordain et al. 2005). The link between maternal nutrition and risk of adult onset noncommunicable disease is now widely accepted. There has been a marked increase in the rates of obesity in women of childbearing age which represents a major issue for both maternal and neonatal health (Compan et al. 2012). Increased body mass index before and/or during pregnancy is strongly associated with a number of direct maternal complications including gestational diabetes, preeclampsia, low‐birth weight, macrosomia, and preterm birth.

Studies have examined the effects of developmental programming in both high maternal salt and of fat independently, associating excessive salt and fat with metabolic disorders, hypertension, and increased risk of cardiovascular events in adult offspring (Howie et al. 2009; Calder et al. 2010; Gray et al. 2013). For the first time, the present study investigates the maternal and developmental programming effects of maternal dietary salt and fat intake in combination. The aim of the present study was to characterize the effect of moderate dietary salt and fat combined on maternal growth and metabolism, fetal and weanling metabolic status and early life growth trajectory in male and female offspring at weaning.

## Methods

### Animal experiments

All procedures described were approved by the Animal Ethics Committee at the University of Auckland (Approval R1069). Eighty Female Sprague–Dawley rats were fed ad‐libitum from weaning until day 90 and maintained at 25°C and a 12 h light: 12 h dark cycle. Female rats were then randomly assigned to four dietary groups and fed ad‐libitum for 21 days prior to pregnancy. Following the prepregnancy habituation period, the experimental groups were fed either (1) Control (CD, *n* = 20) purified standard chow diet (1% NaCl, 10% kcal from fat); (2) 4% Salt diet (SD; 4% NaCl, 10% kcal from fat, *n* = 20); (3) High‐fat diet (HF; 1% NaCl, 45% kcal from fat, *n* = 20) or (4) High‐fat 4% Salt (HFSD; 4% NaCl, 45% kcal from fat) ad‐libitum throughout pregnancy and lactation. Female rats (115 days of age ±2, (*n* = 80)) were time‐mated using an oestrous cycle monitor (Fine Science Tools, North Vancouver, BC, Canada). Day 1 of pregnancy was determined by the presence of spermatozoa after a vaginal lavage and females individually housed thereafter. Food and water intake and body weight of dams were recorded every 2 days. Dams were culled at day 18 of gestation (E18) (*n* = 6/group). Remaining dams were allowed to give birth. All pups were weighed and measured lengthwise, then litter size randomly adjusted to 8 pups (four male, four female) to ensure standardized nutrition until weaning. Pups not allocated to litters were killed by decapitation. All pups were weighed every second day until weaning. Plasma samples were collected and analyzed for insulin, leptin, glucose, and other selected metabolites at all time points. Offspring tissues were collected and weighed at postnatal day 24 (P24, 3 days post weaning). A minimum of 6 L were assessed in parameters relating to offspring.

### Maternal and offspring tissue collection

Blood glucose and ketone levels were obtained from a tail bleed using FreeStyle Optium Blood Glucose and *β*‐Ketone Test Strips (Abbot Diabetes Care Ltd, Victoria, Australia). Trunk blood was collected in lithium heparin 10 mL vacutainers (Becton Dickinson, Auckland, New Zealand) and stored on ice. All blood samples were centrifuged at 748 g at 4°C for 15 min and supernatant stored at −20°C until later analysis. Maternal and offspring tissues were excised, weighed, and stored for later analysis as detailed below.

### Materials

Rat‐specific insulin and leptin ELISAs were sourced from (Crystal Chem Inc, Downers Grove, IL; Catalog #90060 and 90040, respectively). Primers, probes, and TaqMan Universal Mastermix were purchased from Applied Biosystems (ABI, Auckland, New Zealand). All other reagents were purchased from Sigma Aldrich (St. Louis, MO) unless otherwise stated.

### Plasma analysis

Plasma insulin (Crystal Chem Inc), Leptin (Crystal Chem Inc) IL‐1*β*, IL‐6, and TNF*α* concentrations (Quantikine kits; R&D Systems Europe, Abingdon, UK) were measured enzymatically. HOMA‐IR was calculated as [fasting glucose × fasting insulin/22.5]. Maternal E18 and weanling P24 plasma samples were thawed and analyzed for concentrations of free fatty acids (FFA), triglycerides (TAG), low‐density lipoprotein cholesterol (LDL), total cholesterol, high‐density lipoprotein low serum‐3 cholesterol (HDLC3), lactate dehydrogenase, lipoprotein lipase, bilirubin, creatinine, uric acid, alkaline phosphatase (ALP), alanine aminotransferase (ALT), aspartate transaminase (AST), total protein, albumin, and creatine kinase (CK). This analysis was performed using enzymatic colorimetric assays on a Hitachi 902 autoanalyser (Hitachi High Technologies Corporation, Tokyo, Japan).

### Ex vivo adipose tissue culture

Freshly isolated gonadal adipose tissue (AT) was isolated from CD, SD, HF, and HFSD dams at E18. Adipose explants were placed in 24‐well plates (100 mg tissue/well) with 1 mL of complete media (Dulbecco's modified media (DMEM), 10% fetal bovine serum (FBS) and 1% penicillin/streptomycin) for 24 h. Media were harvested and cytokine secretion (TNF*α*, IL‐6, and IL‐1*β*) was analyzed by ELISA (Quantikine kits; R&D Systems Europe).

### Gene expression analysis

RNA was extracted from white AT and liver, using TRI‐Reagent (100 mg tissue/mL) and stored at −80°C. Single‐stranded cDNA was prepared using High‐Capacity cDNA Archive Kit (Applied Biosystems, Warrington, UK). mRNA expression was quantified by real‐time PCR (RT‐PCR) on an ABI 7700 Sequence Detection System (Perkin‐Elmer Applied Biosystems). To control for between‐sample variability, mRNA levels were normalized to the geometric mean of cyclophilinA and hypoxanthine phosphoribosyltransferase (HPRT) for each sample by subtracting the *C*_t_ of controls from the *C*_t_ for the gene of interest producing a Δ*C*_t_ value. The Δ*C*_t_ for each treatment sample was compared to the mean ΔCt for control samples using the relative quantification 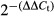 method to determine fold change (Livak and Schmittgen 2001).

### Statistics

Statistical analysis was performed using SigmaPlot for Windows version 12.0 (Systat Software Inc., San Jose, CA). Prepregnancy and pregnancy growth curves and food intakes were analyzed using a repeated measures ANOVA. All the other data were analyzed by two‐way factorial ANOVA, with maternal high fat and maternal high salt intake as factors. Holm–Sidak post hoc tests were performed where indicated to detect further differences between groups. Where data failed normality testing, it was log transformed. Differences between groups were considered significant at *P* < 0.05. All data are presented as means ± SEM unless otherwise stated.

## Results

### Maternal weights and intakes

**Figure 1.**
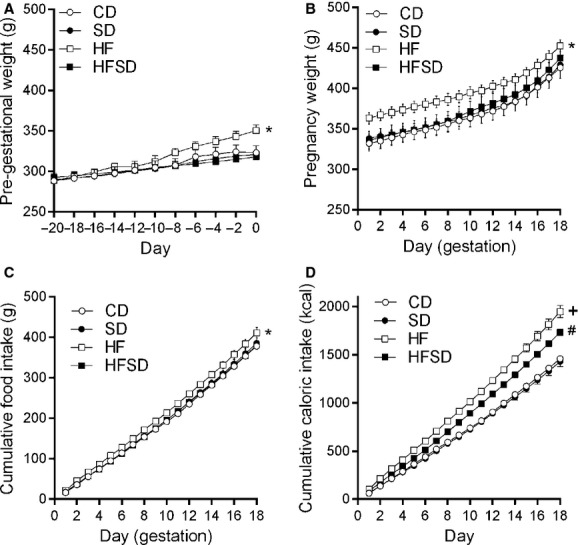
Maternal pregestational and gestational growth curves and food intakes. Female Sprague–Dawley rats were fed either CD (1% NaCl, 10% kcal from fat), SD (4% NaCl, 10% kcal from fat), HF (1% NaCl, 45% kcal from fat), HFSD (4% Salt, 45% kcal from fat) for 3 weeks prior to pregnancy (A) and during pregnancy (B). Body weight was monitored every second day. Cumulative food intakes (C) and total caloric intakes (D) were calculated. Data are presented as means ± SEM. *P < 0.05 for HF versus all other groups; ^+^*P* < 0.05 for HF versus CD and SD; ^#^*P* < 0.05 for HFSD versus CD and SD.

There was a significant increase in prepregnancy body weight in the HF compared to CD, SD, and HFSD females (Fig. 1A). The HF group remained significantly heavier than CD and SD until the end of pregnancy. There was a significant difference between HF and HFSD from day 1 to day 12 of pregnancy (Fig. 1B). No significant difference was observed between HFSD, CD, and SD groups throughout pregnancy (Fig. 1B). HF dams had significantly increased food and kcal consumption throughout pregnancy (Fig. 1C and D). While there was no significant difference in absolute food intake between CD, SD, and HFSD groups, when adjusted for caloric content the HFSD group had significantly increased kcal consumption compared to CD and SD groups (Fig. 1D).

### Maternal adipose deposition and metabolic profile

There was a significant effect of fat on maternal body weight at E18 with significantly increased body weight in HF and HFSD groups compared to CD and SD at the end of pregnancy. While there was no effect of maternal diet on glucose concentration, insulin was significantly increased in the HF group compared to CD and SD. There was no significant difference between HF and HFSD groups. This manifested as an effect of high fat on HOMA‐IR ratios. Ketone concentrations were significantly increased in SD, HF, and HFSD groups compared to CD. As expected, retroperitoneal fat was significantly increased by maternal fat intake, and a significant effect of salt was observed. A significant interaction reflecting reduced retroperitoneal fat in HFSD dams was demonstrated. An effect of fat was observed via significantly increased LDH, Lipase, ALT, and AST concentrations. There was a salt effect on fasting plasma triglycerides concentrations. In addition to altered metabolic parameters, maternal fat consumption led to increased circulating concentrations of proinflammatory cytokines IL‐1*β* and TNF*α* (Table 1).

**Table 1.** Maternal metabolic parameters.

	CD	SD	HF	HFSD
Body weight (g)^+^	418 ± 16.9	449.7 ± 8.8	468.5 ± 9.7^*^	461.9 ± 19^*^
Glucose (mmol/L)	4.75 ± 0.3	4.37 ± 0.3	4.9 ± 0.2	4.83 ± 0.6
Insulin (ng/mL)	1.0 ± 0.2	0.85 ± 0.2	1.7 ± 0.5^*^	1.3 ± 0.2
HOMA‐IR^+^	0.20 ± 0.04	0.15 ± 0.03	0.34 ± 0.08	0.28 ± 0.04
Leptin (ng/mL)	5.67 ± 1.03	4.86 ± 0.78	3.07 ± 1.11	5.82 ± 1.19
Ketone^+^	0.16 ± 0.02	0.33 ± 0.05^*^	0.35 ± 0.02^*^	0.45 ± 0.13^*^
WAT (%BW)^#^^,^^2^	1.14 ± 0.15	1.24 ± 0.11	2.62 ± 0.25^*^	1.58 ± 0.24
TAG (mmol/L)^#^	3.15 ± 0.35	4.56 ± 0.32^*^	3.04 ± 0.37	4.23 ± 0.51^*^
LDH (U/L)	366 ± 64	470.5 ± 45	590.8 ± 79^*^	446.1 ± 26
Lipase (U/L)^+^	10.86 ± 0.6	10.32 ± 0.3	14.03 ± 1.4^*^	12.17 ± 0.5
ALT (U/L)^+^	24.76 ± 4.44	32.17 ± 1.96	38.58 ± 7.32^*^	51.88 ± 6.2^*^
AST (U/L)^+^	102.4 ± 6.7	131.4 ± 6.8^*^	150.4 ± 8.3^*^	152.2 ± 10.8^*^
IL‐1*β* (pg/mL)^+^	8.9 ± 2.4	8.1 ± 1.6	16.5 ± 2.7	12.4 ± 3.5
TNF*α* (pg/mL)^+^	3.7 ± 2.6	2.7 ± 1.7	10.5 ± 3.1^*^	11.3 ± 3.1^*^

Data are mean ± SEM. ^+^*P* < 0.05 for high‐fat diet effect; ^#^*P* < 0.05 for salt effect; ^a^*P *< 0.05 for high fat × high salt interaction, *P < 0.05 w.r.t CD. HOMA‐IR was calculated as [fasting glucose × fasting insulin/22.5]. WAT, white adipose tissue.

### Maternal adipose tissue inflammation

**Figure 2.**
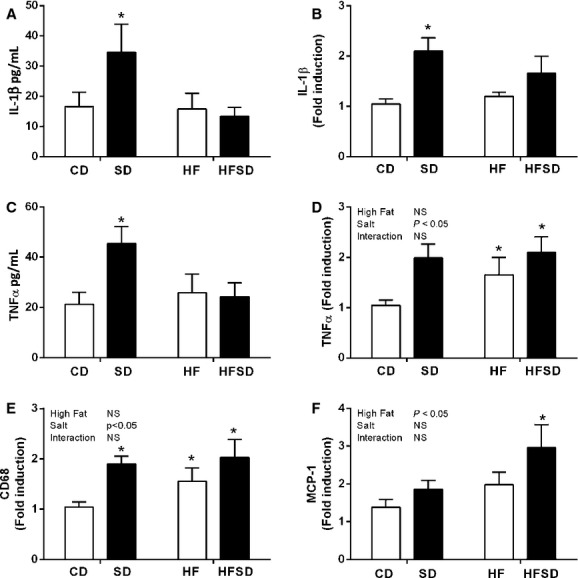
Maternal adipose tissue inflammatory profile. Adipose tissue explants (100 mg/mL) were cultured for 24 h and supernatant was analyzed for (A) IL‐1*β* and (C) TNF*α* using Quantikine ELISA (*P < 0.05, w.r.t CD *n* = 6–7). Adipose tissue mRNA expression of (B) IL‐1*β*, (D) TNF*α*, (E) CD68, and (F) MCP‐1 was analyzed by RT‐PCR (*P < 0.05 w.r.t CD; *n* = 8). Values were expressed as mean ± SEM.

Given increased fat deposition, triglycerides, and circulating cytokine concentrations in our treatment groups, we examined adipose tissue inflammation and function. Adipose explants were cultured for 24 h and cytokine concentrations were assessed. There was significantly increased secretion and expression of IL‐1*β* in the SD compared to CD, HF, and HFSD groups (Fig. 2A and B). While TNF*α* gene expression was significantly elevated in SD, HF and HFSD compared to CD, increased explant TNF*α* secretion was only observed in the SD group (Fig. 2C and D). Interestingly, this increase in proinflammatory cytokines was associated with enhanced expression of CD68, a marker of macrophage infiltration, in SD, HF, and HFSD groups with a significant effect of salt (Fig. 2E). MCP‐1 expression was increased in HFSD but not CD, SD, or HF groups (Fig. 2F).

### Maternal adipose tissue nutrient transport

**Figure 3.**
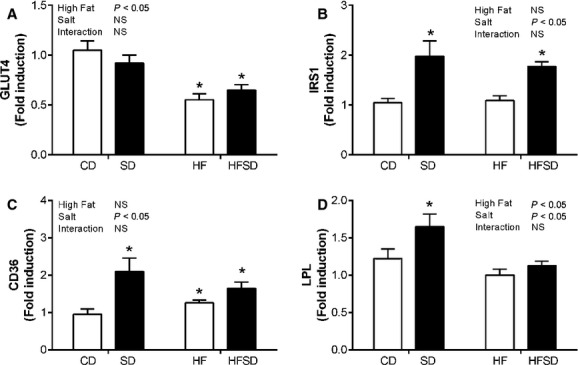
Adipose tissue glucose and lipid homeostasis. Adipose tissue mRNA expression of (A) GLUT4, (B) IRS1, (C) CD36, and (D) LPL was analyzed by RT‐PCR (*P < 0.05 w.r.t CD; *n* = 6–7). Values were expressed as mean ± SEM.

To determine if adipose tissue inflammation had an effect on nutrient transport mediators integral to adipose tissue function, we examined expression of glucose and fatty acid transport. GLUT4 – the main insulin facilitated glucose transporter in adipose tissue – had a significant fat effect with decreased expression in HF and HFSD groups compared to CD and SD (Fig. 3A). However, this did not translate into a reduction in IRS‐1, a major component of the insulin signaling pathway (Fig. 3B). Interestingly there was a significant salt effect on CD36 with significantly increased expression in SD, HF, and HFSD groups compared to CD (Fig. 3C). This was accompanied by significantly increased LPL expression in SD compared to CD, HF, and HFSD groups.

### Effect of maternal diet on adipogenic gene expression

**Figure 4.**
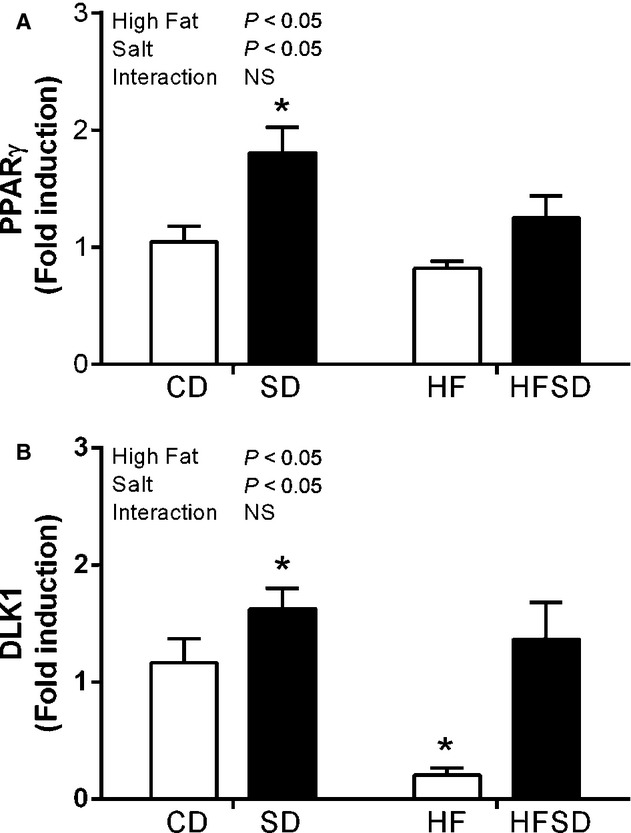
Expression of adipogenic markers. Adipose tissue mRNA expression of (A) PPAR*γ* and (B) DLK1. (*P < 0.05 w.r.t CD; *n* = 6–7). Values were expressed as mean ± SEM.

Given changes in weight gain, adipose tissue inflammation, and nutrient transport, we examined markers relevant to adipogenic pathways. PPAR*γ*, a major regulator of adipogenesis, was significantly increased in the SD groups compared to CD, HF, and HFSD (Fig. 4A). Furthermore, expression of Dlk‐1, a preadipocyte specific marker, was significantly downregulated in HF compared to CD, SD, and HFSD groups (Fig. 4B).

### Effect of maternal diet on hepatic gene expression

**Figure 5.**
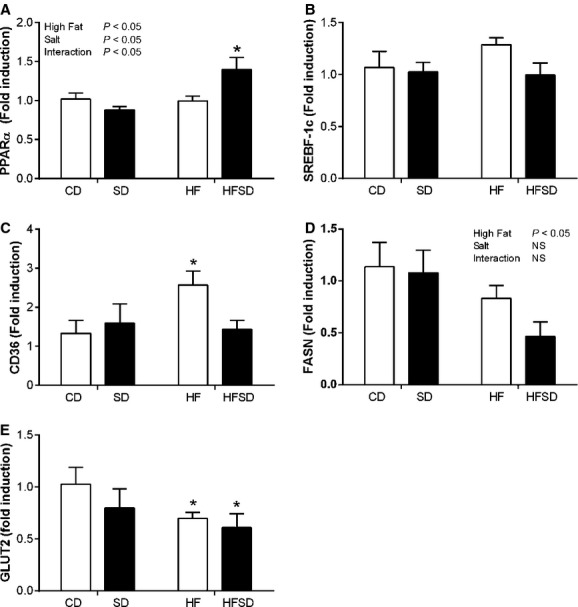
Hepatic metabolic gene expression. Liver mRNA expression of (A) PPAR*α*, (B) SREBP‐1c, (C) FASN, (D) CD36, and (E) GLUT2. (*P < 0.05 w.r.t CD;* n* = 6–7). Values were expressed as mean ± SEM.

Despite significant increases in plasma AST and ALT, there was no significant change in hepatic IL‐1*β* or TNF*α* between dietary groups (data not shown). PPAR*α*, a major regulator of hepatic fatty acid *β*‐oxidation, displayed an effect of fat and salt with significantly increased expression in the HFSD compared to CD, SD, and HF groups (Fig. 5A). There was no significant difference in Srebp‐1c expression between groups (Fig. 5B). There was a significant increase in CD36 in the HF group compared to CD, SD, and HFSD (Fig. 5C). This was accompanied by a fat‐mediated decrease in FASN (Fig. 5D). In addition, there was a significant reduction in GLUT2 expression in the HF and HFSD groups compared to CD (Fig. 5E).

### Effects of maternal diet on fetal body

**Figure 6.**
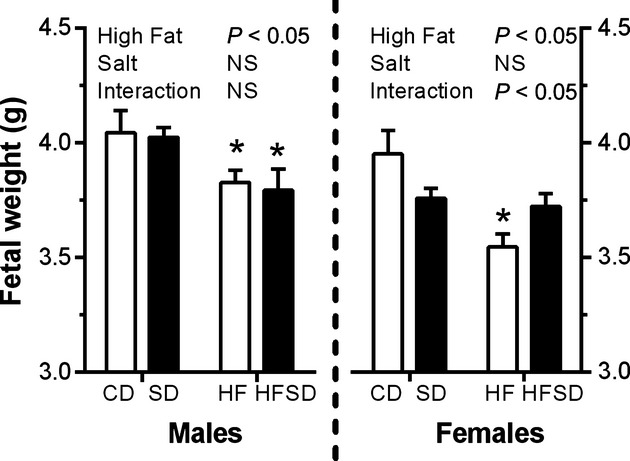
Effects of maternal diet on fetal weight at embryonic day 18. Dams were culled at embryonic day 18. Fetuses were excised and weighed following sex determination. Male and female fetal weights are expressed as mean ± SEM. *P < 0.05 w.r.t CD;* n* = 6–7 L).

Maternal high‐fat diet significantly decreased body weight in both male and female fetuses. In females, a significant interaction was also observed between salt and fat on fetal weight (Fig. 6).

### Effect of maternal diet on weanling growth, physiology, and plasma metabolic profile

**Figure 7.**
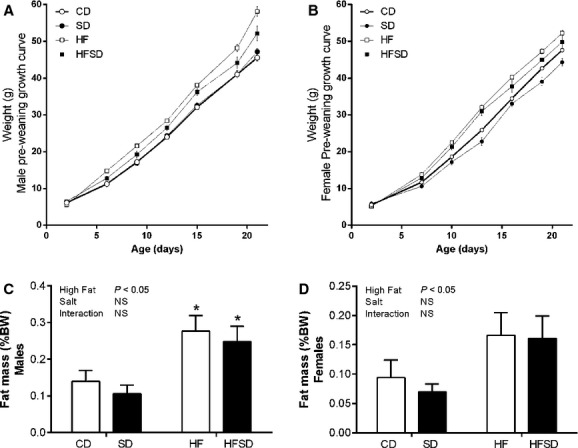
Effect of maternal diet on male and female offspring growth curves and fat deposition. Male growth curves (day 2–21) (A); female growth curves (Hotamisligil and Spiegelman 1994; Bodnar et al. 2005; Cordain et al. 2005; Gual et al. 2005; Chu et al. 2007; Koonen et al. 2007; Berghofer et al. 2008; Challis et al. 2009; Farley et al. 2009; Howie et al. 2009, 2013; Bae et al. 2010; Burri et al. 2010; Calder et al. 2010; Denison et al. 2010; Alfaradhi and Ozanne 2011; Hillemeier et al. 2011; Compan et al. 2012; Gray et al. 2013; Cotechini et al. 2014) (B), male fat deposition expressed as % body weight (C), female fat deposition expressed as % body weight (D). *P < 0.05 w.r.t CD;* n* = 6–7 L). Data are presented as means ± SEM.

Offspring weight was monitored from day 2–21. Both male and female offspring from the HF and HFSD groups displayed significant catch‐up growth and were significantly heavier at the time of weaning compared to CD and SD groups (Fig. 7A and B). This was accompanied by significantly increased fat mass (Fig. 7C and D).

In female offspring, an interaction between salt and fat was observed in blood glucose, plasma insulin, and HOMA‐IR (Table 2). There was a high fat‐induced increase in triglyceride concentrations. SD decreased FFA concentrations. Plasma leptin and ketone concentrations were not different between groups (Table 2).

**Table 2.** Female weanling metabolic profile.

	CD	SD	HF	HFSD
Body weight (g)^+^	52.08 ± 2.2	51.8 ± 1.9	59.75 ± 2.0^*^	53.57 ± 2.6
Glucose (mmol/L)^a^	6.04 ± 0.4	5.69 ± 0.2	5.18 ± 0.3^*^	6.33 ± 0.4
Insulin (ng/mL)^a^	0.75 ± 0.26	0.41 ± 0.09	0.33 ± 0.08	0.69 ± 0.12
HOMA‐IR^a^	0.24 ± 0.11	0.08 ± 0.03	0.1 ± 0.02	0.2 ± 0.04
Leptin (ng/mL)	0.71 ± 0.07	0.68 ± 0.08	0.7 ± 0.08	1.08 ± 0.24
Ketone	2.01 ± 0.27	2.56 ± 0.26	2.78 ± 0.13	2.24 ± 0.38
FFA (mmol/L)^#^	1.21 ± 0.05	0.92 ± 0.05	1.23 ± 0.09	1.7 ± 0.2
TAG (mmol/L)^+^	0.58 ± 0.04	0.60 ± 0.06	0.76 ± 0.07	0.77 ± 0.07

Data are mean ± SEM. ^+^*P *< 0.05 for high‐fat diet effect; ^#^*P *< 0.05 for salt effect; ^a^*P *< 0.05 for high fat × high salt interaction, *P < 0.05 w.r.t CD. HOMA‐IR was calculated as [fasting glucose × fasting insulin/22.5]. *n* = 6–7 L.

In males, there was no significant difference in glucose, insulin, HOMA‐IR, leptin, ketone, or FFA concentrations between maternal dietary groups. An interaction between maternal high‐fat and high‐salt diets was observed in triglyceride concentration as a result of significantly increased concentrations in SD and HF compared to CD groups (Table 3).

**Table 3.** Male weanling metabolic profile.

	CD	SD	HF	HFSD
Body weight (g)^+^^,^^#^	58.25 ± 1.7	54.07 ± 2.3	67.53 ± 2.3^*^	59.1 ± 2.2
Glucose (mmol/L)	5.34 ± 0.17	5.35 ± 0.23	5.6 ± 0.25	5.6 ± 0.42
Insulin (ng/mL)	0.22 ± 0.03	0.36 ± 0.09	0.4 ± 0.12	0.35 ± 0.06
HOMA‐IR	0.056 ± 0.009	0.116 ± 0.065	0.105 ± 0.03	0.099 ± 0.02
Leptin (ng/mL)	0.68 ± 0.09	0.71 ± 0.08	0.76 ± 0.07	0.83 ± 0.18
Ketone	2.84 ± 0.25	2.63 ± 0.21	2.5 ± 0.21	2.93 ± 0.24
FFA (mmol/L)	1.08 ± 0.17	1.12 ± 0.16	1.52 ± 0.22	1.32 ± 0.09
TAG (mmol/L)^a^	0.52 ± 0.07	0.74 ± 0.06^*^	0.83 ± 0.13^*^	0.67 ± 0.05

Data are mean ± SEM. ^+^*P *< 0.05 for high‐fat diet effect; ^#^*P *< 0.05 for salt effect; ^a^*P *< 0.05 for high fat × high salt interaction, *P < 0.05 w.r.t CD. HOMA‐IR was calculated as [fasting glucose × fasting insulin/22.5]. *n* = 6–7 L.

## Discussion

It is increasingly clear that maternal obesity leads to adverse outcomes in offspring (Vickers et al. 2000; Howie et al. 2009; Alfaradhi and Ozanne 2011). In the present study, we investigated the role of moderately high maternal dietary salt and/or fat on maternal metabolic profiles and subsequent consequences for fetal and offspring metabolic homeostasis. We also aimed to determine whether the combination of fat and salt may exacerbate the independent effects of salt and fat. It is clear that developmental programming in offspring is influenced strongly by the timing of maternal nutritional insult (Howie et al. 2013). We, therefore, incorporated an in utero time point, while the developing fetus is directly exposed to an altered maternal nutritional mileau, and a subsequent early postnatal time point to investigate the occurrence of catch‐up growth and initial metabolic dysfunction which may persist throughout life. This study provides evidence that maternal fat and/or salt consumption induces sex‐specific alterations in metabolic parameters synonymous with the development of metabolic disease which may influence the development of adult onset type‐2‐diabetes and obesity in later life.

Throughout pregnancy, increases in hormones such as placental lactogen and growth hormone, prolactin, cortisol, and progesterone inhibit the action of insulin creating a state of insulin resistance, allowing for increased glucose availability for fetal growth. As obesity is also associated with a state of insulin resistance, the maternal diabetogenic environment is enhanced, thereby affecting the growth and development of the fetus. In line with increased body weight, HF dams had increased insulin and HOMA‐IR indices, while the effects were less potent with the HFSD, an intermediate effect was observed with HOMA‐IR. Interestingly, there was a significant salt effect on plasma triglycerides, this was accompanied by increased ketone concentrations across all treatment groups, which indicates a potential shift toward *β*‐oxidation of fatty acids in these dams. This may suggest dysfunctional lipid homeostasis, may have both direct and indirect effects on fetal growth and placental function. Given the evidence of metabolic dysregulation and hyperlipidaemia at a systemic level, we examined the effects of maternal fat and/or salt diets on adipose tissue inflammation and lipid regulation.

Evidence from nonpregnant animal and human studies has linked the progression of adipose tissue dysfunction and insulin resistance to a low‐grade proinflammatory phenotype. Disproportionate lipid accretion from excess caloric intake promotes infiltration of innate immune cells followed by increased secretion of cytokines such as IL‐1*β* and TNF*α* which instigate a chronic inflammatory response resulting in insulin resistance. To date, research on HF‐induced adipose tissue dysfunction centers around nonpregnant individuals. However, there is evidence that inflammatory processes, both local (adipose, placenta) and systemic (circulating cytokines) may be heavily involved in adverse pregnancy outcomes (Denison et al. 2010). Given the pivotal role of IL‐1*β* and TNF*α* in nonpregnancy‐related obesity (Hotamisligil and Spiegelman 1994; McGillicuddy et al. 2011; Reynolds et al. 2012), we examined secretion and gene expression in gonadal fat depots. As per the current literature, there was a significant elevation in HF‐induced TNF*α* expression, a cytokine known to be increased in response to obesity and indeed during pregnancy complications (Cotechini et al. 2014). Interestingly, while IL‐1*β* was not changed in HF dams, there was a significant increase in the SD group. Due to its potency, IL‐1*β* is a tightly regulated protein and requires a two‐step activation process. It is initially produced as a proprotein whose activation is instigated following cleavage of the propeptide by an inflammasome complex. In general, the NLRP3 inflammasome is associated with metabolic inflammation. This inflammasome complex is activated by a series of danger‐associated molecular patterns (DAMPs) which include metabolic stressors such as uric acid, ATP, glucose, and changes in cellular structure such as cell swelling and lysosomal damage (Munoz‐Planillo et al. 2013). There is evidence that high sodium intake upsets intracellular K+ balance thus activating the NLRP3 inflammasome (Compan et al. 2012). While this has not been demonstrated in adipose tissue, we speculate that this may represent a potential mechanism for increased IL‐1*β* in the adipose tissue of SD dams.

Given the extensive remodeling of adipose tissue during pregnancy, it is not surprising that there is a progressive infiltration of macrophages during late pregnancy (Zhang et al. 2011). Furthermore, the proinflammatory changes observed in our treatment groups, we examined whether there was evidence of increased macrophage infiltration into adipose tissue. Indeed, there was a significant increase in CD68 expression, a marker of macrophage activation, in the adipose tissue of each of our treatment groups compared to controls. There was also increased MCP‐1, a macrophage chemoattractant, in the adipose tissue of HFSD dams. While there is evidence of macrophage crown‐like structures in obese pregnant women (Denison et al. 2010) and macrophage infiltration of adipose tissue following HF in nonhuman primates (Farley et al. 2009), to our knowledge this is the first study to link excess salt consumption to adipose tissue inflammation during pregnancy.

In addition to proinflammatory adipose tissue profiles, there were also significant alterations in glucose and lipid homeostasis in fat and/or salt‐fed dams. Despite increased inflammation in the SD animals, there was no evidence of altered GLUT4 expression in adipose tissue. However, in line with increased HOMA‐IR indices, there was a significant reduction in GLUT4 in both HF and HFSD dams. IRS‐1, an important regulator of insulin signaling in adipose tissue, was also examined. Interestingly, despite reduced insulin sensitivity, there was a significant increase in expression in salt‐fed groups. While phosphorylation of IRS‐1 on tyrosine residues is essential for insulin stimulated responses, IRS‐1 phosphorylation on serine residues has the potential to inhibit insulin stimulation (Gual et al. 2005). Ogihara et al. (Ogihara et al. 2001) demonstrated that in nonpregnant animals fed a high‐salt diet, hepatic insulin signaling via IRS‐1 phosphorylation was enhanced despite systemic insulin resistance. However, in the absence of protein data, it is difficult to fully appreciate the physiological impact of salt‐induced increases in IRS‐1 and it is likely that any potential mechanisms pertaining to insulin resistance reside downstream of IRS‐1 signaling.

In the current study, alterations in lipid metabolism and adipogenesis were also observed in SD, HF, and HFSD dams compared to controls. Exposure to salt and/or fat diet during pregnancy increased adipose tissue expression of the fatty acid transporter protein CD36; this appeared to be more potent in the high‐salt dams which also displayed increased lipoprotein lipase expression. Given the propensity of lipid moieties to stimulated toll‐like‐receptors which act as master regulators of innate immune function, increased availability of free fatty acids in the SD and HFSD dams may in part explain enhanced adipose tissue inflammation. Furthermore, PPAR*γ*, which represents an important regulatory factor in adipose tissue lipid storage and indeed adipogenesis, is augmented in the SD dams. This is accompanied by amplified DLK‐1 expression and represents increased preadipocyte numbers with the adipose tissue of SD dams. Together these data suggest that SD dams have an increased preadipocyte pool with the potential for adipocyte differentiation, and therefore enhanced lipogenic capability in these animals. Interestingly, the converse is observed in HF dams, reduced DLK‐1 and PPAR*γ* expression indicates a reduced potential for adipose expansion which may ultimately result in a hypertrophic, insulin‐resistant phenotype.

To further classify the metabolic state of high fat‐ and/or salt‐fed dams we examined hepatic gene expression. Unsurprisingly, HF increases CD36 and decreases GLUT2 expression in line with nonpregnant obese phenotypes (Koonen et al. 2007; Bae et al. 2010). PPAR*α* is a transcription factor which influences hepatic *β*‐oxidation of fatty acids and subsequent ketogenesis (Burri et al. 2010). While there is no difference in CD, SD, and HF PPAR*α* expression, a significant interaction between salt and fat accompanied by a decrease in GLUT2 expression indicates a potential shift toward hepatic *β*‐oxidation of fatty acids in HFSD dams. Interestingly, we observe elevated plasma ketone concentrations in SD and HF dams with a significant fat/salt interaction in HFSD dams. Ketones cross the placenta and have detrimental effects on fetal growth and neonatal brain structure (Sussman et al. 2013), therefore, HFSD diets may represent a link between dietary exposure to salt and/or fat and adverse fetal outcomes.

There is significant evidence to support maternal obesity and HF‐induced developmental programming of offspring metabolic dysfunction. Interestingly, the impact of high‐salt diets are not as well established and the combination of maternal salt and fat intake has yet to be investigated in the context of developmental programming, despite their respective prevalence in Western‐style diets. Given the greatly increased inflammatory and metabolic disturbances, observed both locally (adipose and liver) and systemically, our aim was to ascertain whether this resulted in altered fetal and neonatal growth and basic metabolic profiles. Indeed, male and female fetuses from HF and HFSD had significantly retarded growth at day 18 of gestation. Salt diets alone had only a minor effect on male fetal growth but induced significant growth restriction in female fetuses. Offspring were monitored until the end of weaning and HF and HFSD male and female offspring exhibited catch‐up growth associated with significantly enhanced fat mass which is commonly linked to maternal HF‐induced programming (Vickers 2011). Despite these effects, there are no effects between groups in either male or female offspring on glucose homeostasis as measures by HOMA‐IR. This is perhaps unsurprising given the age and lack of secondary dietary insult to these animals. However there is evidence, even at this early postweaning time point that there are perturbations in plasma lipid profiles, however, this only occurs in female offspring.

The mechanisms involved in the progression of developmental programming leading to adult metabolic disease remain poorly understood. The current study provides further evidence that increased maternal consumption of fat and salt independently and, for the first time, in combination, negatively impacts the developing offspring's growth trajectory and potentiates risk factors for metabolic disorders during later life. These results also emphasize the importance of a nutritionally balanced diet when pregnant with significant detrimental effects on inflammation and metabolism in adipose tissue and liver and indeed systemically, which may persist postpregnancy thus predisposing mothers to future metabolic complications. The ability of maternal metabolic factors such as inflammatory cytokines and ketone bodies to cross the placenta can have serious consequences on fetal and neonatal growth and development. This model offers a robust nutritional paradigm in which mechanistic aspects of maternal metabolism and developmental programming may be usefully and carefully explored. Based upon the data presented, we would echo the recent commentaries and initiatives to reduce both the quantity of salt and fat currently in a typical ‘Western diet’.

## Acknowledgments

The authors wish to express their gratitude for the help and support provided by the VJU, Rachna Patel, Angelica Bernal, and Minglan Li. All work in this manuscript was performed at the Liggins Institute, Grafton Campus, University of Auckland.

## Conflict of Interest

None declared.
